# Food-Specific Inhibitory Control Mediates the Effect of Disgust Sensitivity on Body Mass Index

**DOI:** 10.3389/fpsyg.2019.02391

**Published:** 2019-10-22

**Authors:** Xing Liu, Ji Li, Ofir Turel, Rui Chen, Qinghua He

**Affiliations:** ^1^Faculty of Psychology, Southwest University, Chongqing, China; ^2^Information Systems and Decision Sciences, California State University, Fullerton, CA, United States; ^3^Key Laboratory of Mental Health, Institute of Psychology, Chinese Academy of Sciences, Beijing, China; ^4^Chongqing Collaborative Innovation Center for Brain Science, Southwest University, Chongqing, China

**Keywords:** disgust sensitivity, body mass index, obesity, inhibition control, mediation

## Abstract

Disgust is an emotion that drives food avoidance. People vary in their responses to disgust, which is captured by their disgust sensitivity. Disgust sensitivity is clinically significant because it can influence eating behaviors, and indirectly people’s body mass index (BMI). Inhibitory control can also influence BMI through the role that such reflective abilities play in governing food intake. In this study, we relied on neural models of disgust to suggest that disgust and inhibitory control are intertwined, and that inhibitory control facilitates the translation of disgust sensitivity into BMI. Mediation analyses applied to 46 subjects, including 29 normal body weight [BMI = 18.34 kg/m^2^ (SD = 1.58)] and 17 overweight/obese [BMI = 26.03 kg/m^2^ (SD = 2.58)] subjects, were used to test the hypothesis. Subjects completed the Chinese version of the Disgust Scale-Revised, and an inhibition control test (Food-Specific Stop-Signal Task). There were negative correlations between the disgust sensitivity score (DS) and body mass index (BMI), and between DS and stop-signal reaction time (SSRT). Moreover, BMI was positively correlated with SSRT. The mediation model results showed that disgust sensitivity was associated with BMI and that this relationship was mediated *via* inhibition control. There was no significant effect of DS on BMI, while the effect of SSRT on BMI was significant. This suggested that the effect of disgust sensitivity on BMI was fully mediated through food-specific inhibitory control. This supports our hypothesis that BMI is affected by disgust sensitivity and that this relationship is mediated by inhibition control. These findings reveal a key mechanism that underlies disgust sensitivity-BMI association and point to future research and potential interventions aimed at food intake management.

## Introduction

Disgust is an emotion that captures revulsion at the prospect of oral incorporation of offensive objects (Rozin and Fallon, 1987). It is an important protective mechanism that discourages humans from consuming things that could harm them; it ultimately prevents contamination and disease spread (Rozin et al., 1999). It also serves other avoidance behaviors, such as inhibiting the breaking of social norms (Pond et al., 2012). Disgust sensitivity is the trait-like predisposition of a person to become disgusted in response to a particular group of stimuli, known as disgust elicitors (Merckelbach et al., 1999). Disgust sensitivity is clinically significant because it can help individuals avoid external dangerous stimuli; however, individuals high in disgust sensitivity are prone to anxiety, fear of death, and unnecessarily avoiding risk-taking (Haidt et al., 1994). As such, disgust sensitivity can be associated with a variety of psychiatric disorders, including eating disorders (Aharoni and Hertz, 2012; Ammann et al., 2018), phobias (Bianchi and Carter, 2012), and obsessive-compulsive disorders (Ólafsson et al., 2013).

It has been demonstrated that disgust sensitivity is associated with eating disorders and body mass index (BMI), which is a reliable measure of body fat content (Troop et al., 2000; Griffiths and Troop, 2006; Mayer et al., 2008; Davey and Chapman, 2009; Houben et al., 2013; Watkins et al., 2015; Vicario and Rafal, 2017; Ammann et al., 2018). For example, Troop et al. (2000) and Mayer et al. (2008) found that eating disorder symptoms (as captured with thinness and bulimia scores) were related to higher levels of disgust sensitivity, including to food. Similarly, Davey and Chapman (2009) showed that the experience of disgust may be heightened in individuals with eating disorder symptomatology; and Houben and Havermans (2012) found that more restrained eaters have increased core disgust, increased contamination disgust, and a decreased appetite for high-calorie food, compared to women with higher BMIs.

Importantly, disgust is rooted in brain activity in the insular cortex (i.e., the insula) (Wicker et al., 2003; Wright et al., 2004; Chen et al., 2009), especially in its anterior parts (Krolak-Salmon et al., 2003; Jabbi et al., 2008). The sensitivity of the insula to stimuli can vary among individuals, which makes it a potential contributor to disgust sensitivity (Schienle et al., 2005; Stark et al., 2005; Calder et al., 2007; Mataix-Cols et al., 2008; Schafer et al., 2009). Indeed, it has been shown that the insula has decreased activation in an obese group as compared with a lean group when viewing contaminated food images (Watkins et al., 2015). Nevertheless, the effect of disgust sensitivity on behavior inhibition (including presumably food intake inhibition) is likely indirect, as inhibition tasks are executed in the prefrontal cortex (Shackman et al., 2009; Stramaccia et al., 2015). Indeed, the insula has trajectories, especially from its superior anterior parts to the prefrontal cortex (Flynn et al., 1999; Droutman et al., 2015), which afford disturbing self-control tasks by hijacking the prefrontal cortex (Naqvi et al., 2007; Craig, 2009; Naqvi and Bechara, 2009, 2010; Turel et al., 2018). Therefore, it is reasonable to assume that disgust sensitivity, which is manifested through insular activity, indirectly exerts an effect on eating behaviors, through its effect on self-control abilities, which manifest in prefrontal cortex activity.

Inhibitory control regulates eating behavior (Allom and Mullan, 2014; Carbine et al., 2017, 2018; Zhou et al., 2018), because it helps people make flexible choices in the face of changing environments (Miyake et al., 2000; Lavagnino et al., 2016) and help resisting impulsions toward reward-generating behaviors that are sub-optimal (Turel and Bechara, 2016; Turel and Qahri-Saremi, 2016, 2018; He et al., 2017a,b, 2018; Chen et al., 2018). Indeed, less effective inhibition control is associated with increased food intake and overeating, increased weight and obesity, and lower weight loss during treatment (Houben et al., 2013; Giel et al., 2017; Oomen et al., 2018). In the context of food intake, the stop-signal task is an effective means to capture food-specific inhibitory control (Bartholdy et al., 2016). Inhibitory control is significantly impaired in obese adults and children compared to individuals with normal body weight (Lavagnino et al., 2016). From a brain activation standpoint, Hendrick et al. (2012) observed that obese females had lower activity in the insula, inferior parietal cortex, cuneus, and supplementary motor area compared to lean females. Furthermore, the brain activations in these regions inversely correlated with BMI scores. As such, inhibitory control ability is critical for the regulation of food intake and may therefore influence body weight.

Synthesizing these views and relying on the neural model according to which insular activity, which mediates disgust, affects behavior through trajectories to brain centers that govern inhibitory control (Naqvi et al., 2007, 2014; Naqvi and Bechara, 2009, 2010; He et al., 2014; Droutman et al., 2015; Turel et al., 2018), we hypothesize that the effect of disgust sensitivity on body mass index is mediated through inhibitory control.

## Materials and Methods

### Participants

We recruited 52 participants (34 females, all university students) *via* class announcements. Their height and weight were measured; these were used for BMI calculation (weight to squared height in kg/m^2^). There were 18 overweight (BMI > 23, averaged BMI = 26.14 ± 2.61, 13 females) and 34 normal weight subjects (BMI < 23, averaged BMI = 19.27 ± 1.60, 22 females). All subjects were right-handed, with normal or corrected-to-normal vision. They had no self-reported history of neurological or psychiatric disorders. Six participants were excluded for further analysis because they did not follow the directions of the stop-signal tasks. This left us with 46 participants, out of which 17 were overweight (BMI = 26.03 ± 2.58, 12 females) and 29 were normal weight (BMI = 18.34 ± 1.58, 20 females). The ratio of overweight to normal weight participants in this sample is consistent with previous large sample studies of Chinese young adults (He et al., 2015). The study procedure was reviewed and approved by the Institutional Review Board of Southwest University (SWU-IRB-17-031).

### Measures

#### Disgust Scale-Revised Chinese Version

The scale was derived from the modified Disgust Scale-Revised (DS-R) (Olatunji et al., 2009). DS-R has 25 items along three dimensions: a 12-item core disgust scale, an 8-item animal-reminder scale, and a 5-item contamination disgust scale. Core disgust is a basic disgust elicitor. It focuses on disgust related to oral consumption of offensive items such as rotting foods. Animal reminder disgust is based on reminding people of their own animalistic nature; examples include disgust associated with corpses. Lastly, contamination disgust relates to interpersonal contagion that reflects threat of disease transmission from other people (Olatunji et al., 2008; Hamerman, 2016). DS-R has been shown to be valid and reliable across cultures and languages (Olatunji et al., 2009). The translation of the DS-R to Chinese followed a forward-backward translation procedure by four bilingual psychology professionals. It also included adjustments for cultural differences. The final iteration produced a scale that presumably accurately reflects the context-adjusted English items of DS-R.

We used this scale in test-retest procedure with 520 college students (328 females, averaged age = 21.4 ± 2.24 years), and found that the scale was reliable (Cronbach alpha coefficient, total scale = 0.82, core disgust subscale = 0.76, animal-reminder subscale = 0.87, contamination disgust subscale = 0.91) and valid (confirmatory factor analyses fit indices: *χ*^2^/df = 3.76, RMR = 0.06, GFI = 0.92, AGFI = 0.94, NFI = 0.92, TLI = 0.91, CFI = 0.91, RMSEA = 0.04). There was a moderate correlation between the three sub-dimensions, with correlation coefficients ranging from 0.34 to 0.53 (all with *p* < 0.05); there were high correlations between the sub-dimension scores and the total score (correlation coefficients are 0.78, 0.74, and 0.87, respectively, all with *p* < 0.001). We therefore used the total score of the Chinese DS-R (DS-RC) as a measure of disgust sensitivity. Differences in DS-RC scores among gender, grade, region, and BMI groups in the sample are given in Table 1. Consistent with prior research, obese individuals had lower disgust sensitivity scores.

**Table 1:** Differences between groups in the sample in disgust sensitivity.

Covariate	Disgust sensitivity			
	Mean	SD	*t*	*p*
Gender				
Male (*n* = 252) Female (*n* = 268)	50.64 61.45	17.60 14.14	−7.798	<0.001
Grade				
Freshman (*n* = 120) Others (*n* = 400)	57.88 55.71	15.41 17.17	1.372	0.269
Region				
Rural (*n* = 310) Urban (*n* = 210)	56.84 55.26	16.11 17.75	0.958	0.289
BMI				
BMI > 24 (*n* = 71) BMI < 24 (*n* = 449)	46.07 57.72	17.06 16.37	5.377	<0.001

##### Food-Specific Stop-Signal Task

The food-specific stop-signal task was adapted from the general Stop-Signal Task (SST) to measure response inhibition specifically for food. The task flow is consistent with the traditional SST, and the only difference is that the background pictures involve food items. These pictures were selected from the Chinese Affective Picture System. The high- and low-calorie food images were matched in terms of arousal and valence, and this was verified with a panel of Chinese students.

**Figure 1:**
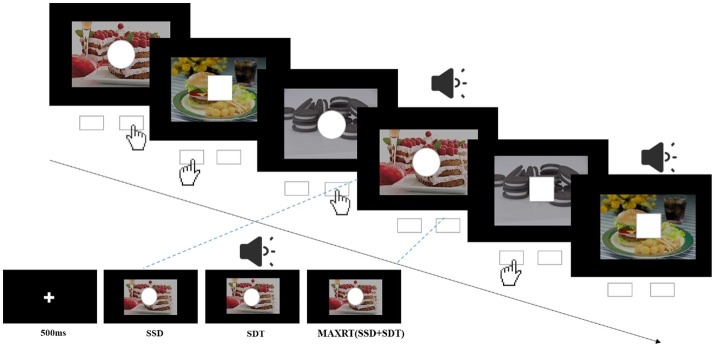
Illustration of the stop-signal task. For go trials, subjects were instructed to press left key when the stimulus was a square and right key when it was a circle. For a quarter of all trials, the go stimulus was followed by an auditory stop signal (pure tone) after a variable stop-signal delay (SSD). SDT, duration of the stop signal; MAXRT, maximum reaction time.

The task was generated by Stop-it software (Verbruggen et al., 2008). After a 500-ms fixation cross on the black screen, a stimulus in the form of either square (1 cm side length) or circle (1 cm radius) appeared against the background of either high- and low-calorie food images (Figure 1). The subjects were asked to press the left or right response key (counterbalanced across participants) for the square or circle as fast as possible. Participants were instructed not to respond when the sound stimuli was presented (the stop signal, 75-Hz pure tone, lasted for 75 ms). The stop signal was presented in 25% of the trials, consistent with the traditional SST. The delay between the go stimulus and the stop signal (stop-signal delay, SSD) was initially set at 250 ms and was subsequently dynamically changed using a tracking procedure to enable participants to correctly inhibit 50% of the stop trials: following a successful inhibition, the SSD was increased by 50 ms; while following a failed inhibition, the SSD was decreased by 50 ms. The dependent variable, stop-signal reaction time (SSRT), was calculated by subtracting the mean SSD from mean reaction times. Higher SSRTs indicate decreased inhibitory control (Wessel and Aron, 2015). Each subject completed four blocks of the task, and each block consisted of 64 trials. Before the experiment, each subject completed one practice block. The process is depicted in Figure 1.

### Procedure

In order to reduce the effect of hunger on the study, the experiment was uniformly arranged between 12:00~14:00 and 17:30~19:30, which was right after meals (lunch and dinner breaks, respectively). In addition, each participant rated his or her hunger level on a 1 (not hungry at all) to 10 (very hungry) scale. All participants completed the DS-RC, followed by the food-specific stop-signal task, and the measurement of their height and weight.

## Results

### Descriptive Statistics of Variables

**Figure 2:**
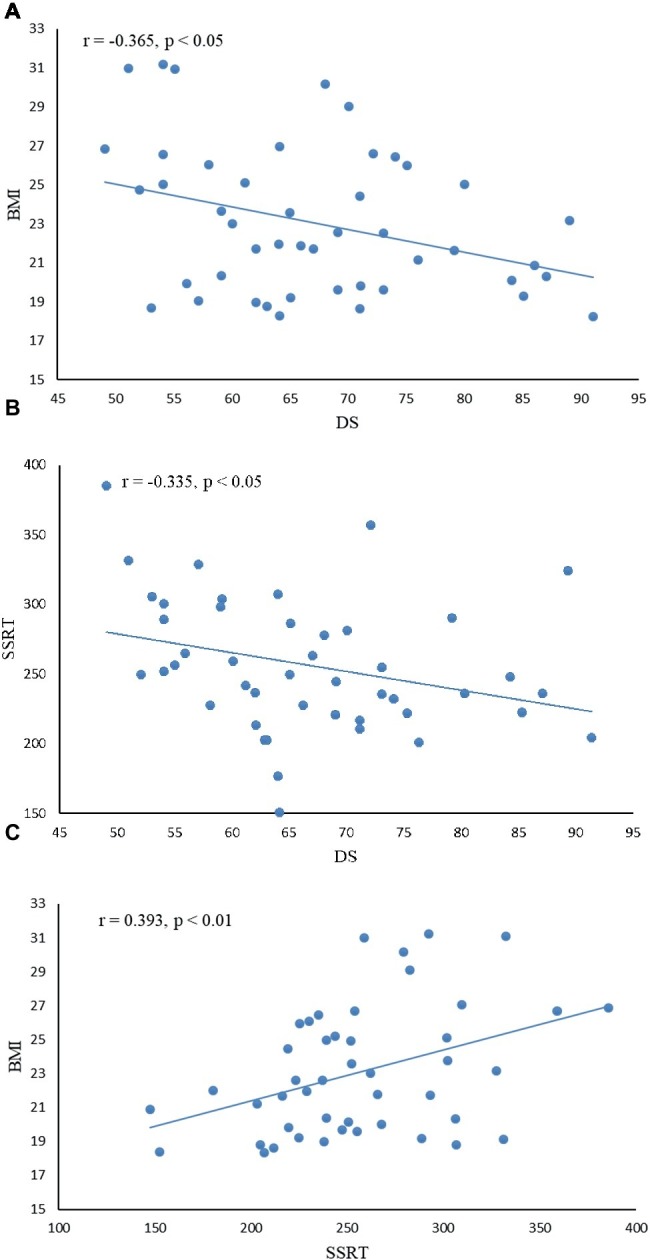
Correlation between disgust sensitivity score (DS), inhibition control index (SSRT), and body mass index (BMI). **(A)** There was a negative correlation between DS and BMI (*r* = −0.365, *p* < 0.05), indicating that subjects with low disgust sensitivity had high BMI. **(B)** DS was negatively correlated with SSRT (*r* = −0.335, *p* < 0.05), indicating that subjects with low disgust sensitivity had poor control ability. **(C)** BMI was positively correlated with SSRT (*r* = 0.393, *p* < 0.01), indicating that higher BMI subjects had poorer inhibitory control.

Descriptive statistics and correlations among disgust sensitivity score (DS), body mass index (BMI), and stop-signal reaction time (SSRT) are given in Table 2; correlations are further depicted in Figure 2. There was a negative correlation between DS and BMI and between DS and SSRT, indicating that subjects with low disgust sensitivity had high BMI and poor control ability. BMI was positively correlated with SSRT, indicating that higher BMI subjects had poorer inhibitory control compared to normal-BMI subjects.

**Table 2:** Descriptive statistics.

	*M*	SD	BMI	SSRT
Disgust sensitivity score (DS)	62.52	10.74	−0.37^*^	−0.34^*^
Body mass index (BMI)	22.19	3.75	–	0.39^**^
Stop signal reaction time (SSRT)	253.72	58.74	–	–

** p < 0.01

* *p < 0.05*.

**Figure 3:**
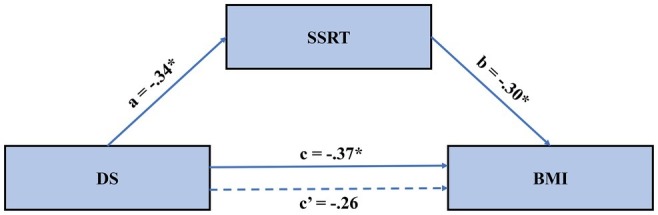
The mediated model of the effect of disgust sensitivity on BMI. Results suggested that the effect of disgust sensitivity on BMI is fully mediated through food-specific inhibitory control. BMI, body mass index; DS, disgust sensitivity; SSRT, stop-signal reaction time.

### Hypothesis Testing

We followed common mediation testing procedures (Baron and Kenny, 1986) with the PROCESS macro in SPPS (Hayes, 2013) to test our hypothesis. In these analyses, disgust sensitivity was the independent variable, obesity degree was the dependent variable, and inhibition control was the mediator variable. First, DS, BMI, and SSRT were mean-centered. Second, the effects of centered DS on BMI were examined with a regression model. The results showed that DS significantly reduces BMI (Table 3). Third, the effect of centered DS on SSRT was examined with a regression model. As shown in Table 3, DS significantly reduced SSRT on the food-specific SST. Lastly, the joint predictive effect of DS and SSRT on BMI was examined; see Model C in Table 3; the mediated model is further depicted in Figure 3. Results showed no significant effect of DS on BMI, while the effect of SSRT on BMI was significant. This suggests full mediation. That is, the effect of disgust sensitivity on BMI was fully mediated through food-specific inhibitory control. This lends support to our hypothesis and the neural model of disgust translation into food consumption behavior. Accounting for both DS and SSRT explained 22% of the variance in BMI, suggesting that these two factors are important but not exclusive determinants of BMI.

**Table 3:** Mediation tests.

	Independent variable	*β*	SE	*t*	*F*	*R*^2^
Model A (BMI)	DS	−0.37	0.14	−2.60^*^	6.78^*^	0.13
Model B (SSRT)	DS	−0.34	0.14	−2.36^*^	5.58^*^	0.11
Model C (BMI)	DS	−0.26	0.14	−1.84	5.93^**^	0.22
	SSRT	0.30	0.14	2.13^*^		

** p < 0.01

* *p < 0.05*.

## Discussion

This study sought to unravel a key mechanism through which disgust sensitivity affects food intake and indirectly, BMI. To theoretically develop this mechanism, we relied on insights from the neurocognitive science literature that suggest that the insula, which is a center for processing disgust, exerts influence on behavior *via* its trajectory to the prefrontal cortex, which is a center for inhibitory control. Based on these insights, we suggested that inhibitory control mediates the effect of disgust sensitivity on BMI. Our results support a full mediation and lend support for this perspective.

These results reaffirm the findings in previous studies in which disgust sensitivity scores were negatively associated with BMI; and participants with lower DS had a significantly higher BMI compared to participants with a higher DS (Houben et al., 2013; Watkins et al., 2015). This by itself is important because BMI abnormalities have many adverse consequences. For example, excessively high BMI is associated with a higher risk of diabetes; cardiovascular, heart disease; hypertension; stroke; and sleep apnea (Narayan et al., 2007; Freedman and Sherry, 2009; Qizilbash et al., 2015; Turel et al., 2016). Similarly, excessively low BMI in anorexic individuals can adversely affect people’s health and normal functioning (Bulik et al., 2005; Nickols-Richardson, 2008; Sanford, 2017). Our Model A (Table 3) shows that DS alone explains 13% of the variance in BMI. Thus, it can be an important intervention target for scholars and professionals trying to help obese and overweight populations to return their BMIs to a normal desirable range.

Our result extends this view and explains one facet of how this association works. Specifically, it demonstrates that one key mechanism through which disgust sensitivity influences BMI is through a reduction in inhibitory control that accompanies increases in disgust sensitivity. This perspective provides another layer of support, albeit behavioral, to the neurocognitive perspective of decision-making. According to this perspective, the insula mediates interoceptive signals such as disgust, and these hijack prefrontal cortex activity and prevent proper inhibition (Naqvi et al., 2007, 2014; Naqvi and Bechara, 2009, 2010; Clark et al., 2014; Droutman et al., 2015; Turel et al., 2018). These findings also corroborate previous evidence linking inhibition control with BMI (Guerrieri et al., 2009; Hendrick et al., 2012; Lavagnino et al., 2016). Thus, future research may further focus on the insula-prefrontal cortex interaction, and/or on inhibition-disgust interactions as a means to examine and intervene in BMI abnormalities. The mediation model with both disgust sensitivity and food-specific inhibitory control (Model C in Table 3) explained 22% of the variance in BMI. Thus, disgust sensitivity and food-specific inhibitory control can be important metrics and targets in future research on interventions aimed at restoring BMI normality.

Several limitations of this study are noteworthy. First, the Chinese version of Disgust Scale-Revised (DS-RC) used in this study had good reliability and validity; however, it may fail to consider the cultural differences between China and the west. Disgust sensitivity can be culturally dependent (what may be seen as disgusting in one culture may not be perceived as such in another). Thus, the social context in which an individual is located may affect his or her experience of disgust stimuli. Therefore, we call for future research to consider a Chinese-specific disgust sensitivity scale that reflects common Chinese taste preferences, cultural elements, and social conditions, and to examine the generalizability of our findings to other cultures. Second, while we relied on a neurocognitive model to develop the hypothesis, we used only behavioral and anthropometric measures in this study. Even though the link between the insula and prefrontal cortex has been demonstrated (Droutman et al., 2015), and the understanding of the neural basis of responses to gustatory and food stimuli has evolved (Yeung et al., 2019), future research should corroborate the link between brain activation and our measures. Third, we focused on a limited set of predictors of obesity for theoretical and simplicity reasons. Future research can expand our model to account for more predictors of obesity (e.g., genetics, family environment). Lastly, this study used a small sample and the participants were not excessively abnormal in terms of BMI. This may impact the generalizability of the findings. Future research should therefore replicate our results with samples containing excessively high BMI (morbidly obese) and excessively low BMI (e.g., anorexic) participants.

## Conclusions

This is the first study to our knowledge to use the Disgust Scale-Revised Chinese Version (DS-RC) to examine individual differences in disgust sensitivity and to explore the relationship between disgust sensitivity and obesity. The results showed that the DS-RC had high reliability and validity and was suitable for the measurement of Chinese college students’ disgust sensitivity. Consistent with prior research, individuals with low disgust sensitivity had a high BMI and poor control ability to suppress the desire to eat food, and higher BMI individuals had poorer inhibitory control compared to normal-BMI individuals. This study innovatively used inhibition control as a mediator variable, to examine the predictive effect of disgust sensitivity on obesity. We found that the effect of disgust sensitivity on BMI is fully mediated through food-specific inhibitory control.

## Data Availability Statement

The datasets generated for this study are available on request to the corresponding author.

## Ethics Statement

The studies involving human participants were reviewed and approved by Institutional Review Board of Southwest University. The patients/participants provided their written informed consent to participate in this study.

## Author Contributions

JL and QH designed the study. JL and RC performed the study. XL, JL, and QH analyzed the data. XL, JL, OT, RC, and QH wrote the manuscript. All authors made final approval of the draft submitted to the journal.

### Conflict of Interest

The authors declare that the research was conducted in the absence of any commercial or financial relationships that could be construed as a potential conflict of interest.
